# Cross-Sectional Serological Survey of Human Fascioliasis in Canutama Municipality in Western Amazon, Brazil

**DOI:** 10.1155/2018/6823638

**Published:** 2018-02-08

**Authors:** Marcel Gonçalves Maciel, Walter dos Santos Lima, Francisco Lazaro Moreira de Almeida, Leila Inês Aguiar Raposo Câmara Coelho, Guilherme Alfredo Novelino Araújo, Mariana Gomes Lima, Luiz Henrique Gonçalves Maciel, Cíntia Aparecida de Jesus Pereira, Thaís Costa da Silva Maciel, Jorge Augusto de Oliveira Guerra, Rosa Amélia Gonçalves Santana, Maria das Graças Vale Barbosa Guerra

**Affiliations:** ^1^Programa de Pós-Graduação em Medicina Tropical/Universidade do Estado do Amazonas/Fundação de Medicina Tropical Dr. Heitor Vieira Dourado, Av. Carvalho Leal 1777, Cachoeirinha, 69065-130 Manaus, AM, Brazil; ^2^Universidade Federal de Minas Gerais (UFMG), Av. Pres. Antônio Carlos 6627, Pampulha, 31270-90 Belo Horizonte, MG, Brazil; ^3^Secretaria Municipal de Saúde de Canutama (SEMSA), Avenida Benjamin Constant 929, Centro, 69820-000 Canutama, AM, Brazil; ^4^Universidade Federal do Amazonas (UFAM), Av. General Rodrigo Octavio Jordão Ramos 1200, Coroado I, 69067-005 Manaus, AM, Brazil; ^5^Laboratório Central de Saúde Pública do Estado do Amazonas (LACEN), Rua Emílio Moreira 528, Centro, 69020-040 Manaus, AM, Brazil; ^6^Universidade Federal Rural do Rio de Janeiro (UFRRJ), Rodovia BR 465, Km 07, s/n, Zona Rural, 23890-000 Seropédica, RJ, Brazil; ^7^Faculdade Metropolitana de Manaus (FAMETRO), Av. Constantino Nery, 69050-010 Manaus, AM, Brazil; ^8^Fundação de Medicina Tropical Dr. Heitor Vieira Dourado (FMTHVD), Av. Pedro Teixeira N. 25, Dom Pedro, 69040-000 Manaus, AM, Brazil

## Abstract

**Background:**

Fascioliasis is an important parasitic disease. In the northern region of Brazil, a human parasite infection has been reported through a coprological survey. Eggs of* Fasciola hepatica* were found in fecal samples of 11 individuals. Knowledge of the infection in animals or the presence of snails is necessary to address the possibility of the parasite cycle occurrence in that region. The aim of this study was to describe the transmission of human fascioliasis in Canutama, Amazonas, in Western Amazonia, Brazil.

**Methods:**

Serological (ELISA and Western Blot, WB) and parasitological analyses were carried out in humans. In addition, the presence of the intermediate snail host within the community was examined.

**Results:**

A total of 434 human samples were included in the study, of which 36 (8.3%) were reactive by ELISA and 8 (1.8%) were reactive by WB.* Fasciola hepatica* eggs were found in one human sample. The occurrence of the intermediated host was recorded and 31/43 specimens were identified as* Lymnaea columella. Conclusion.* Canutama constitutes a focus of transmission of human fascioliasis. This study describes the first serological survey for human fascioliasis, as well as its simultaneous occurrence in human hosts and possible intermediates performed in northern Brazil.

## 1. Introduction

Fascioliasis is an important zoonotic helminthiasis with a great impact on human development, a good example of an emerging/reemerging parasitic disease in many countries as a consequence of environmental change, and man-made modifications. Two digenean trematode species,* Fasciola hepatica* and* Fasciola gigantica*, infect a wide variety of domestic animals and humans to cause hepatic parasitosis [1].

According to estimates, 180 million people in 75 countries in the world are at risk of infection, and this parasitic disease is included among the major neglected tropical diseases [2, 3]. Furthermore, fascioliasis affects around 600 million animals on several continents, causing damage to the health of the herds, contributing to the decrease in milk production, loss of carcass weight, and death of animals, and resulting in economic losses estimated at up to 2 billion dollars per year in South and North America [4].

The transmission of this parasite to its definitive host occurs after the ingestion of water and food contaminated with infective forms of the parasite, usually wild watercress* (Nasturtium officinale)* or grass on which metacercariae are encysted. The parasite cycle depends on the existence of intermediate hosts an mollusks of the family Lymnaeidae, genus* Lymnaea *[5]. In most human cases fascioliasis is asymptomatic although nonspecific symptoms such as fever, abdominal pain, diarrhea, and nausea occur which may be related to acute infection or chronic infection [6].

The diagnosis of infection in humans can be performed by a combination of a direct method such as examination of eggs in the feces of the individual or with an indirect method such as serological tests that identify IgG or IgM class antibodies against the parasite [7]. Imaging exams such as computed tomography, magnetic resonance imaging, and abdominal ultrasonography are also used for diagnosis of chronic cases [8].

In Brazil, the first registry of this parasitosis in humans occurred in 1958 in a patient in the State of Mato Grosso [9], with additional cases diagnosed and described later mainly in southern and southeastern states [10, 11]. In the northern region of Brazil, the human parasite infection was reported in 2005, identified by a coprological survey in Canutama, South of the State of Amazonas. Eggs of* Fasciola hepatica* were found in the fecal samples of 11 individuals [12]. This study describes the first serological survey for human fascioliasis in northern Brazil.

## 2. Materials and Methods

### 2.1. Study Area

**Figure 1:**
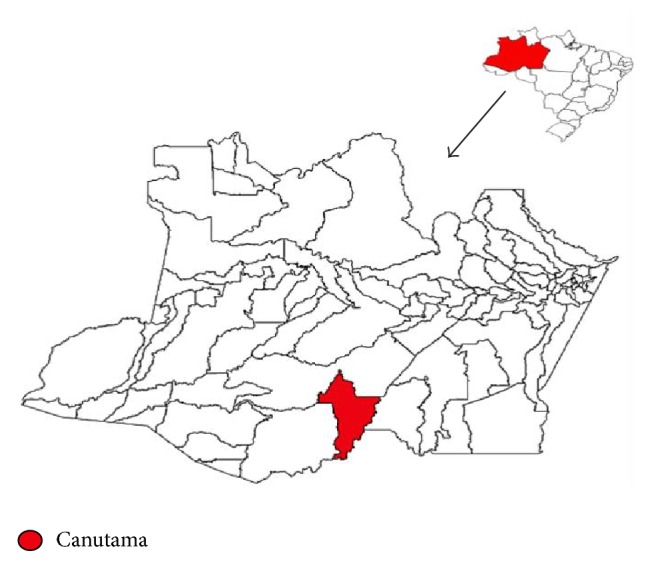
*Study area*. The map of the State of Amazonas (bottom), in Brazil (top right corner), shows the municipality of Canutama in red.

The study was conducted in the municipality of Canutama located 55 m above sea level in the Purus river, one of the largest rivers in the world. Canutama is located on the South of the State of Amazonas, 620 km from the capital Manaus, with geographic coordinates S06°32′19.7 W064°23′22.7. The monthly meteorological mean of precipitation is 186 mm, humidity 86.1%, and temperature 32.7°C (Figure 1).

### 2.2. Human Sample Size Calculation

The sample was calculated taking into account a sample error of 5% and a confidence interval of 95% for a population of 12,727 people and a 2% infection for a sample of 434 people.

### 2.3. Recruitment of Participants and Ethic Statements

The serological and coprological investigations of the studied population were carried out in August 2013, selecting one in six houses in a floodplain area in the urban section of the city, including samples of individuals from 2 a 86 years of age. Participants under 18 years of age were enrolled in the study only after authorization from their respective parents, and all participants signed the Free and Informed Consent Form. This study was approved by the Brazilian National Ethics Committee Board, Opinion number 319.463.

### 2.4. Serological Survey

In the serological survey, 10 mL sample of blood was collected from each patient and centrifuged in the laboratory in Canutama. The serum was frozen and sent to the Central Laboratory of Public Health of Amazonas (LACEN/AM), in Manaus, to perform the ELISA serological test (DRG International) for the identification of anti-*Fasciola hepatica* IgG antibodies. The test is based on excretion/secretion antigens of* F. hepatica* predominantly containing fluke cysteine proteases. All steps were carried out according to the manufacturer's instructions. Human sera, negative and positive controls, and cut-off control (CO) (1 : 100 dilution) were added to triplicate wells. Plates were read after 15 minutes on a multiscan ELISA plate reader at an absorbance of 450 nm. The test run may be considered valid if substrate blank has an absorbance value below 0,100, negative control below 0,200, CO control between 0,250 and 0,750, and positive control above 0,600. The results are given as the mean of the optical density (OD) obtained from triplicate samples expressed as a percentage of the CO, using the following formula: Percent  positive  (PP) = (Mean  OD  of  test  sample)/(Mean  OD  of  C+)/100. A serum is considered positive when its absorbance value is above 10% of CO. The results in DRG Units (DU) were calculated according to the following formula: DU = (sample  (mean)  absorbance  value × 10)/CO. The results were negative if DU < 9 and positive if DU > 11 [13].

The samples with reactive or indeterminate results were submitted to the Western Blot (LDBIO Diagnostics), performed with strips and reagents provided with the kit, according to the manufacturer's instructions. Briefly, the strips were incubated with serum diluted to 1 : 50 in Tris-NaCl sample buffer for 90 min. After a washing step with Tris-NaCl washing buffer, the strips were incubated with an antihuman immunoglobulin G-alkaline phosphatase conjugate for 60 min. After another washing step, the protein fractions recognized by the serum were revealed by the corresponding substrate-chromogenic solution containing nitroblue tetrazolium and 5-bromo-4-chloro-3-indolyl phosphate. The reaction was stopped by washing the strips with distilled water. The strips were dried and glued to paper for reading and storage. A test was considered to be positive when the strip presented 2 or more specific bands including the P27-28 kDa band. Positive and negative controls were tested in each assay [14]. Test was performed at the Laboratory of Immunology of the Entomology Center of the Tropical Medicine Foundation of Amazonas, Dr. Heitor Vieira Dourado (FMTHVD).

### 2.5. Coprological Survey in Humans

To investigate* Fasciola hepatica* egg and other parasites in the feces of studied population Lutz [15] method was carried out in two phases. First, the fecal samples of all participants of the serological survey were included, processed, and analyzed in the Municipal Laboratory of Clinical Analysis of the Municipal Health Department of Canutama. In the second phase only stool samples with reactive serological result were studied at the Multidisciplinary Laboratory of the Dr. Heitor Vieira Dourado Tropical Medicine Foundation in Manaus.

### 2.6. Malacological Research

A sample containing 43 specimens of snails was stored in 50 ml polypropylene tubes containing 25 mL of absolute alcohol. After fixation the sample was sent to the National Reference Laboratory in Schistosomiasis, Malacology (FIOCRUZ/RJ), responsible for registration and identification of the material based on observation of the shells and morphological aspects performed using a binocular stereoscope microscope.

### 2.7. Statistical Analysis

The fascioliasis prevalence in the municipality was calculated. A level of alpha significance of 0.05 and a confidence coefficient of 95% were set. The association between patients with reactive and nonreactive serology for fascioliasis and qualitative variables (sociodemographic and exposure) was evaluated using Fisher's Exact Test. In order to identify the relationship between the proportions, the Odds Ratio was calculated. The statistical software used in the analysis was SPSS (Statistical Package for the Social Sciences) version 16.0 and program R 3.2.2.

## 3. Results

A total of 434 participants were included in the study, 260 (50%) females and 181 (42%) in the age group 0–19 years. More than half (256 or 59%) reported having a primary school education, all (100%) natural of the State of Amazonas and none had moved to another state or municipality in the last two years. According to the information reported by participants, 433 (99.8%) lived in houses with walls and floors of wood, 427 (98.4%) had houses covered with zinc tiles, 408 (94%) enjoyed running water, 404 (93.1%) did not have water piping and stored water in containers such as basin and buckets, 428 (98.6%) had no sewage network, and 287 (66.1%) dumped their waste in streams, ditches, streams, or rivers (Table 1).

Regarding dietary habits, 284 (65.4%) reported consuming raw foods such as lettuce, green odor, coriander, and tomato, and 376 (86.6%) said they grew these foods around their homes in raised beds. When these factors were associated with human infection by fascioliasis there was no statistical significance. None of the participants had heard about fascioliasis or its transmission dynamics or knew about the role of snails in the life cycle of the parasite or in maintaining the disease; 6 (1.4%) stated that herbivorous animals such as cattle, sheep, goats, and horses were raised near their homes; 307 (70.7%) reported having snails in the vicinity of their homes and statistical significance was found in the variable occupancy (*P* = 0.012) for individuals involved in agricultural activities (Table 1).

### 3.1. Human Fascioliasis Tests

**Figure 2:**
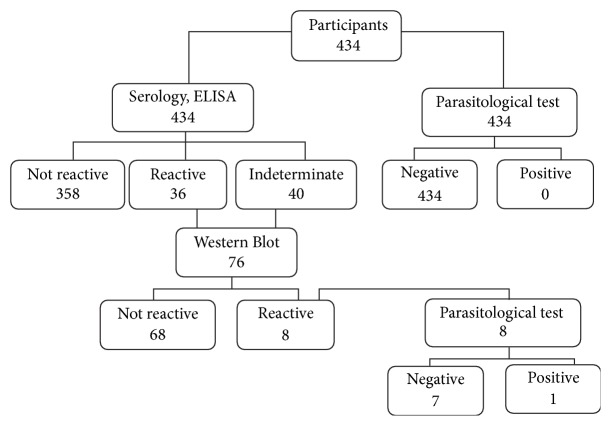
Flowchart of the survey and tests performed in the study population.

Of the 434 samples, 36/434 (8.3%) were reactive and 40 (9.2%) inconclusive in the ELISA test. These 76 samples were submitted to WB test; the immunodominant bands most recognized were 27-28 kDa (100%) and 42 kDa (100%), in which 8 (2%) were reactive for* Fasciola hepatica *infection (Table 2), with a general prevalence of 1.8% (IC95% = 0.8–3.6) (Figure 2). The average age was 41 years; 5 (62.5%) were male, 4 (50%) were illiterate, and 5 (62.4%) (*P* = 0.012) worked in agriculture. All of them lived in houses with walls, ceilings, and wooden floors and consumed treated water; 6 (75%) consumed raw vegetables such as lettuce, green odor, coriander, and tomato. All of them reported having animals (cattle, goats, and pigs) and 5 (62.5%) snails in the surroundings of their houses (Table 1).

**Figure 3:**
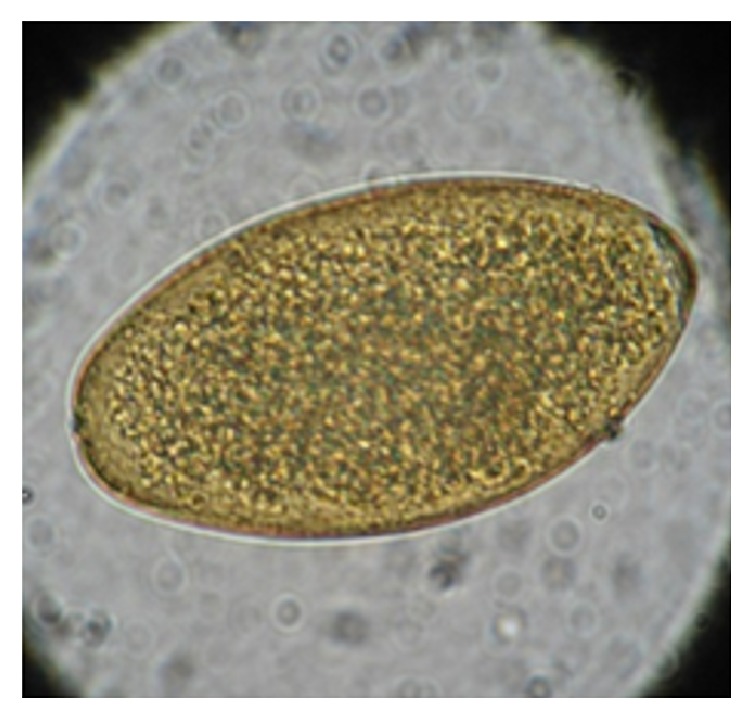
The image of an egg from* Fasciola hepatica* found in fecal material of one participant is shown as seen under the microscope at 400x magnification; its size was 143 *μ*m by 75 *μ*m.

No samples were positive for* Fasciola hepatica *in the first phase of the coprological survey. Only* Ascaris lumbricoides* were observed, in the second phase, fecal samples were analyzed from 8 serology-reactive individuals, and in 1/8 (12.5%) a* Fasciola hepatica* egg was found measuring 143 *μ*m in length and 75 *μ*m in width (Figure 3).

### 3.2. Clinical Evaluation

All 8 seropositive patients were submitted to clinical examination, and 3 (37.5%) patients reported abdominal pain, 2 (25%) reported feeling nauseous, 4 (50%) reported having fever, 2 (25%) complained of weight loss, 2 (25%) complained of fatigue, and 3 (37%) reported occasional episodes of diarrhea (Table 2).

### 3.3. Malacological Research

**Figure 4:**
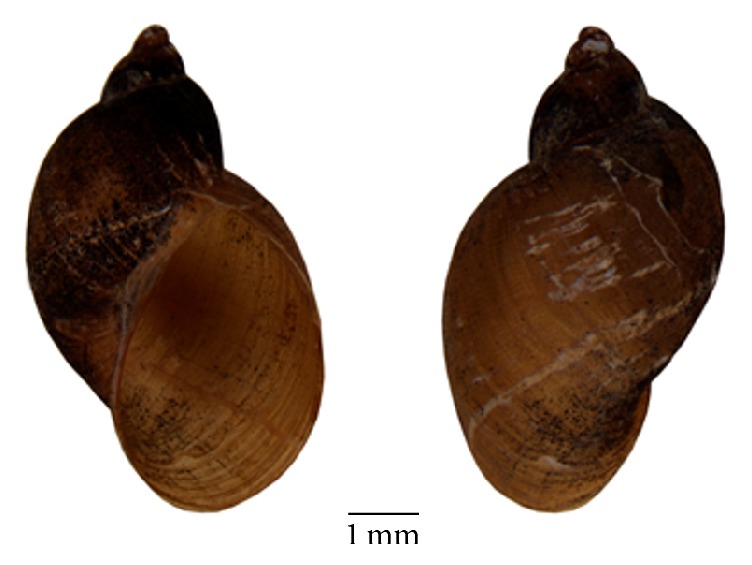
A picture of* Lymnaea columella * collected at study area.

Of the 43 specimens sent for identification, 31 were identified as* Lymnaea columella*, presenting coil winding in a spiral and absence of teeth in the columellar wall of the opening, 9 specimens were identified as* Physa marmorata*, presenting coil winding in a sinistral spiral, and 3 specimens were identified as* Succinea* spp. (Figure 4). A picture of* Lymnaea columella *was collected at study area.

## 4. Discussion

In the present study we describe the results of the first serological survey for human fascioliasis in Brazil including the first registry of intermediate hosts where reactive individuals were found in northern Brazil. Although the first record of* Fasciola hepatica* eggs in Brazilian populations was reported in 1958 [11], in the northern region with the exception of Canutama there are still no fascioliasis records, which explains the lack of knowledge of the population about this disease shown here.

Until the end of the 1990s, human fascioliasis was considered a secondary disease. This has changed significantly in recent years, when the World Health Organization (WHO) became aware of reports from different countries indicating that fascioliasis infection in humans was probably more frequent than previously accepted, in South, Central, and North America, as well as in Europe, Africa, and Asia [3, 5]. However, the traditional concept of a disease of sheep and cows still remains. Diagnostic limitations and the fact that human fascioliasis is not a disease of obligatory declaration suggest that the number of human cases is higher than that published [7]. The ELISA test (DRG International) has been used for diagnosis in endemic areas, with sensitivity up to 99,5% [13]. Its use associated with parasitological examination has been proposed as a better screening alternative [7] to reduce the possibility of false negative results compared to only parasitological examination of feces.

Because Western Blot is a serological test with high specificity for fascioliasis diagnosis based on parasite-specific secretion-excretion proteins [14] it was used to confirm reactive and undetermined ELISA test results or to rule out false positive results due to cross reactions. Although coprological examination is considered a gold standard for human fascioliasis diagnosis, this method has limitations. It is not always possible to find* Fasciola hepatica* eggs in the feces of infected individuals [7], as shown in this study, where only one of the 8 seropositive participants was positive parasitological examination. In endemic area in the Bandar-Anzali, from 1,984 volunteers 9 of the 30 seropositive persons were egg positive [16].

In these areas, both children and females are most severely affected. Although the disease seems to affect mainly children, detection of fascioliasis infection in various age groups has been reported in studies conducted in countries of South America and the Middle East [17, 18]. In this study the reactive population was composed of adults, but in a previous study in this locality this parasitosis was reported in children up to 5 years old [12], insertion agreement with this municipality fitting the mesoendemic classification.

Poor sanitation and outdoor defecation, observed in the study population, contribute to transmission intensity in mesoendemic areas [19]. Canutama is a poor municipality lacking basic sanitation and a sewerage system. Fecal waste is dumped into small streams and ditches that flow into streams and rivers surrounding the municipality, leading to water contamination with the feces of* Fasciola hepatica *infected individuals. Inadequate storage and treatment of drinking water along with certain habits of the population can favor fascioliasis and other parasitosis as observed in this study and in countries neighboring Brazil, such as Peru and Bolivia which are considered hyperendemic for fascioliasis [20–22].

In the Amazonas state, there are reports of* Lymnaea columella *in 6 of the 62 municipalities in floodplain areas as is common in the Amazon: Benjamin Constant, Careiro, Coari, Manaus, Tefé, and Iranduba [23]. The presence of* Lymnaea columella* near participants homes throughout the year supports autochthony of this parasitosis in Canutama and can contribute to the maintenance of this disease during low tide and spread during high tide, representing a risk to the riverine population. The study areas were in floodplain environments, subject to the flood and ebullient periods of the Purus river that create emergence and maintenance of snail populations.

In dietary habits of study population greens are consumed raw in sauces or as an accompaniment to fish, a common staple. According to the population, small snails are found in their flowerbeds feeding on vegetables, mainly chives* (Allium schoenoprasum)*. It is important that people know that human fascioliasis may be prevented by implementing strict control of watercress and other metacercariae carrying aquatic plants for human consumption, especially in endemic zones [24].

This study describes the occurrence of fasciolosis transmission in area of low altitude which is in agreement with reports in similar areas (55 m above sea level) [5, 25] and shows that this parasitosis is dispersing to a new area in Brazil, although this dispersion had been predicted. The ability of* Fasciola *to spread is related to its ability to colonise and adapt to new environments, despite adverse conditions at very high altitude such as the Bolivian Altiplano [26].

Although in the northern region of Brazil only the municipality of Canutama has registered this parasitosis, neighboring countries have been classified as endemic for fascioliasis [21, 22, 26, 27]. In addition, the presence of* Lymnaea columella* in 7 state municipalities [23] may contribute to the dispersion and maintenance of the parasite cycle and consequently of the disease in the region. The adaptation of* Fasciola hepatica* and its intermediate hosts at different temperatures, humidity, precipitation levels, and altitudes highlight a need for surveillance by public health agencies, as well as support by development agencies to carry out new investigations into the occurrence of human disease in other municipalities of the state.

## 5. Conclusion

There is a transmission focus of human fascioliasis in Canutama. Autochthonous cases of fascioliasis were identified in diagnosed patients without a history of travel to other cities.

Identified factors such as poor knowledge about the disease by the local population, lack of information regarding the parasite's transmission dynamics, and the presence of the intermediate host* Lymnaea columella* suggest that the number of human cases infected in this locality is being underestimated and that there is underreporting of cases.

Therefore, there is a need for extensive surveys including a study of ruminants to identify possible parasite reservoirs as well as monitoring of intermediate hosts to address infection rate and the adoption of prevention strategies and disease control in the region.

## Figures and Tables

**Table 1:** Demographic and socioeconomic characteristics in the study population.

Category/subcategory	Reactive *N* (%)	Not reactive *N* (%)	Total *N* (%)	Odds ratio	IC (95%)	*P* value
*Gender*						
Female	3 (1.2)	257 (98.8)	260 (59.9)			0.276
Male	5 (2.9)	169 (97.1)	174 (40.1)			
*Age (years)*						
0–19	0 (0)	181 (100)	181 (41.7)			0.144
20–39	5 (3.4)	141 (96.6)	146 (33.6)			
40–59	2 (2.6)	76 (97.4)	78 (18)			
41–59	1 (3.4)	28 (96.6)	29 (6.7)			
*Education*						
Illiterate	4 (3.6)	106 (96.4)	110 (25.3)			0.323
Primary school	3 (1.2)	253 (98.8)	256 (59)			
Secondary school	1 (1.5)	65 (98.5)	66 (15.2)			
University	0 (0)	2 (100)	2 (0.5)			
*Occupation*						
Retired	0 (0)	19 (100)	19 (4.4)			0.012
Public agent	1 (5.9)	16 (94.1)	17 (3.9)			
Student	0	179 (100)	179 (41.2)			
Farmer	5 (7.1)	65 (92.9)	70 (16.1)			
Fisherman	0 (0)	19 (100)	19 (4.4)			
Self-employed	0 (0)	13 (100)	13 (3.0)			
Domestic	2 (1.7)	115 (98.3)	117 (27)			
*Type of housing*						
Brick	0 (0)	1 (100)	1 (0.2)		0.0	>0.99
Wood	8 (1.8)	425 (98.2)	433 (99.8)			
*Floor in residences*						
Cement	0 (0)	1 (100)	1 (0.2)		0.0	>0.99
wood	8 (1.8)	425 (98.2)	433 (99.8)			
*Type of roof*						
Asbestos	0 (0)	7 (100)	7 (1.6)		(0.00–43.19)	>0.99
Aluminum	8 (1.9)	419 (98.1)	427 (98.4)			
*Piped water*						
No	1 (3.8)	25 (96.2)	26 (6.0)	2.26	(0.05–18.96)	0.393
Yes	7 (1.7)	401 (98.3)	408 (94)			
*Water treatment*						
No	8 (2.0)	426 (98)	434 (100)		0.0	>0.99
Yes	0	0	0			
*Water storage*						
Bowl/bucket	0 (0)	30 (100)	30 (6.9)		0.0	>0.99
Others	8 (2.0)	396 (98)	404 (93.1)			
*Sewage network*						
No	8 (1.9)	426 (98.1)	434 (100)		0.0	>0.99
Yes	0 (0)	0	0			
*Waste dump*						
Stream, ditch, river, bayou	7 (2.4)	280 (97.6)	287 (66.1)	33.64	(0.46–165.5)	0.275
Black trench	1 (0.7)	146 (99.3)	147 (33.9)			
*Animals in peridomestic *						
Cattle, sheep, goats, horses, and pigs	0 (0)	6 (100)	6 (1.4)		0.0	>0.99
No	8 (1.9)	420 (98.1)	428 (98.6)			
*Snails in peridomestic*						
No	3 (2.4)	124 (97.6)	127 (29.3)	1.46	(0.22–7.63)	0.697
Yes	5 (1.6)	302 (98.4)	307 (70.7)			
*Consumption of raw vegetables*						
No	2 (1.3)	148 (98.7)	150 (34.6)	0.63	(0.06–3.56)	0.72
Yes	6 (2.1)	278 (97.9)	284 (65.4)			
*Planting of greenery*						
On the ground	0 (0)	58 (100)	58 (13.4)	0.0	(0.00–3.83)	0.605
Suspended	8 (2.1)	368 (97.9)	376 (86.6)			
*Heard about fascioliasis*						
No	8 (1.9)	426 (98.1)	434 (100)		0.0	>0.99
Yes	0 (0)	0 (0)	0 (0)			

**Table 2:** Test results and symptoms reported by participants with reactive serology.

	Reactive participants							
	1	2	3	4	5	6	7	8
Gender	Male	Male	Female	Male	Male	Female	Female	Male
Age	69	58	55	37	35	30	27	23
ELISA	+	I	+	+	+	+	I	+
WB (P27-P28 kDa, P42 kDa)	+	+	+	+	+	+	+	+
*Fasciola hepatica *	−	−	−	−	+	−	−	−
*Ascaris lumbricoides*	−	−	−	+	−	−	+	−
Abdominal pain	**+**	**−**	**+**	**+**	**−**	**−**	**−**	**−**
Diarrhea	**+**	**−**	**+**	**+**	**−**	**−**	**−**	**−**
Fatigue	**−**	**−**	**−**	**+**	**−**	**+**	**−**	**−**
Fever	**+**	**−**	**+**	**+**	**−**	**+**	**−**	**−**
Nausea	**+**	**−**	**−**	**+**	**−**	**−**	**−**	**−**
Weight loss	**+**	**−**	**+**	**+**	**−**	**+**	**−**	**−**
